# Continuous Spectrum LEDs Promote Seedling Quality Traits and Performance of *Quercus ithaburensis* var. *macrolepis*

**DOI:** 10.3389/fpls.2017.00188

**Published:** 2017-02-14

**Authors:** Sonia Smirnakou, Theoharis Ouzounis, Kalliopi M. Radoglou

**Affiliations:** ^1^Department of Forestry and Management of the Environment and Natural Resources, Democritus University of ThraceNea Orestiada, Greece; ^2^Horticulture and Product Physiology Group, Department of Plant Sciences, Wageningen UniversityWageningen, Netherlands

**Keywords:** *Quercus* species, continuous LED spectrum, controlled environment, seedling quality, nursery performance

## Abstract

Regulation of the growth, development, and quality of plants by the control of light quality has attracted extensive attention worldwide. The aim of this study was to examine the effects of continuous LED spectrum for indoor plant pre-cultivation and to investigate the morphological and physiological responses of a common broadleaved tree species in Mediterranean environment, *Quercus ithaburensis* var. *macrolepis* at seedling developmental stage. Thus, the seedlings were pre-cultivated for 28 days, under five different LED light qualities: (1) Fluorescent (FL) as control light (2) L20AP67 (high in green and moderate in far-red), (3) AP673L (high in green and red), (4) G2 (highest in red and far-red), AP67 (high in blue, red, and far-red), and (5) NS1 (highest in blue and green and lowest in far-red) LEDs. Further examination was held at the nursery for 1 year, on several seedling quality traits. Indeed, AP67 and AP673L triggered higher leaf formation, while L20AP67 positively affected seedling shoot development. NS1 and AP67 LED pre-cultivated seedlings showed significantly higher root fibrosity than those of FL light. Furthermore, NS1 and AP673L LEDs induced fourfold increase on seedling root dry weight than FL light. Hence, evaluating the seedling nursery performance attributes, most of those photomorphogenetic responses previously obtained were still detectable. Even more so, LED pre-cultivated seedlings showed higher survival and faster growth indicating better adaptation even under natural light conditions, a fact further reinforced by the significantly higher Dickson’s quality index acquired. In conclusion, the goal of each nursery management program is the production of high quality seedlings with those desirable traits, which in turn satisfy the specific needs for a particular reforestation site. Thus, the enhanced oak seedling quality traits formed under continuous LEDs spectrum especially of NS1 and AP673L pre-cultivation may potentially fulfill this goal.

## Introduction

Among the various environmental factors that affect plant growth and development, light is the main impetus for the plant life cycle (Stuefer and Huber, 1998). Changes in light quality, are perceived by plants through different types of photoreceptors, including phytochromes (red and near infrared wavelengths), and cryptochromes, phototropins, and Zeitlupes (blue and ultraviolet-A wavelengths) (Jiao et al., 2007; Galvão and Fankhauser, 2015; Huché-Thélier et al., 2016). Also the photoreceptor absorbing ultraviolet-B has been identified as UVR8 recently (Rizzini et al., 2011). The phytochrome responses vary with plant species, cultivar, age, irradiance, spectral quality and temperature; for instance, low levels of far-red light in the spectrum or a high ratio between red and far-red commonly result in short, compact poinsettia plants (*Euphorbia pulcherrima* Willd. ex Klotzsch) (Mata and Botto, 2009). Furthermore, cryptochromes are known to affect stem extension, and a variety of plants respond to blue light by suppressing shoot elongation (Oyaert et al., 1999). However, the opposite effect with increased shoot elongation under pure blue light compared to red light has also been reported in a number of species such as Salvia (*Salvia splendens* F. Sello ex Ruem and Schult. cv. Red Vista) and marigold (*Tagetes erecta* L. cv. Orange Boy) (Heo et al., 2002). Roots, which usually grow underground and are not exposed to light, also possess photoreceptors such as phytochromes at relatively high levels (Tóth et al., 2001; Casal, 2012); specifically phytochromes A, B, and D have been shown to control red light-mediated elongation of the primary root (Correll and Kiss, 2005). In addition, red light may promote the synthesis of chlorophylls (Ma et al., 2001), while blue light may induce changes in stomatal development, density and opening, increases leaf area and decreases chlorophyll synthesis (Liu-Gitz et al., 2000); green light affects various plant growth and developmental processes (Folta and Maruhnich, 2007) such as stomata opening and photosynthesis (Terashima et al., 2009). Moreover, UV-B irradiation has also been reported to cause different responses with respect to growth, production of dry matter and physiological and biochemical changes to plants (Mpoloka, 2008; Fedina et al., 2010). Some plant species are unaffected by UV-B irradiation while in several growth is enhanced, but most species are sensitive and prone to damage, such as rice and maize (Du et al., 2011; Lidon, 2012). Thus, all the aforementioned photoreceptors affect various physiological processes.

Fluorescent lamps are generally used as a conventional light source for growing plants in indoor cultivation, however, the light from these lamps contain unwanted wavelengths that are inadequate in promoting growth and there is still limitation in their ability to control light quality (Ohashi-Kaneko et al., 2007). On the other hand, LEDs had been considered as a better alternative with improved features such as a smaller mass and volume, a longer life, relatively cool emitting surface, lower power requirement and an effective single wavelength for morphogenesis and photosynthesis (Zukauskas et al., 2002; Bourget, 2008). In recent years, the use of LEDs as a radiation source for plants has attracted considerable interest because of its vast potential for developmental and photomorphogenetic studies as well as for its commercial applications (Bian et al., 2015; Yeh et al., 2015). Thus, the selection of an optimal light source is an essential task in closed-type plant production systems, which are fully reliant on artificial light sources.

The demand for bigger, better, and faster-growing seedlings has been ever-growing; as a result, forest seedling production is a continually evolving technology in reforestation (Villar-Salvador et al., 2004). Poor oak regeneration (Smit et al., 2009) and high adult mortality in dry or degraded areas constitute a major concern regarding the implementation of artificial regeneration programs (Cortina et al., 2004). Direct seeding of oak species is generally considered as a non-preferable method due to water limitations (Leyva and Fernández-Alés, 1998), animal predation (Johnson et al., 2002), or easily loss of recalcitrant acorn viability when humidity drops bellow high levels (Villar-Salvador et al., 2013). Oaks are generally considered to be less sensitive to drought because of deep-penetrating roots, xeromorphic leaf structure and effective stomatal control of transpirational water loss (Kubiske and Abrams, 1993). However, drought tolerance may differ considerably among oak species and provenances, reflecting adaptation to environments with varying water availability (Dickson and Tomlinson, 1996). Consequently at nursery level, the production of seedlings in containers considered as a more reliable regeneration method (Cortina et al., 2004), by noticeably increased growth, mainly in terms of their root systems (Aphalo and Rikala, 2003; Domínguez-Lerena et al., 2006). Seedlings with more roots have better survival potential and better establishment capability after field planting (Davis and Jacobs, 2005; Tsakaldimi et al., 2009). On harsh sites, a greater capability of water and nutrient absorption and transport from roots through the stem to the transpiring shoot system give seedlings a better chance to overcome planting stress (Grossnickle, 2005). However, seedling plantations often show poor results in dry and semi-arid areas, especially regarding Mediterranean evergreen oaks and other broadleaved sclerophyllus species, due to low seedling quality (Valdecantos et al., 2006). Therefore, evaluating seedling quality is crucial for understanding seedling development in the nursery, as well as the subsequent field growth and survival (Ward et al., 2000).

*Quercus ithaburensis* is an East-Mediterranean deciduous oak, where the sub-species macrolepis is present in Greece according to Fotiadis et al. (2009). During the last decades, activities such as conversion of forests to agricultural land, illegal lumbering and overgrazing have confined *Q. ithaburensis* var. *macrolepis* to small-forested patches or to isolated individuals in the interior of forested islets in lowland and semi-mountainous agricultural fields (Pantera et al., 2008; Pantera and Papanastasis, 2012). In the past few years, there has been a growing interest for the species to be included in reforestation as well as in restoration projects (Tsakaldimi et al., 2000; Tsitsoni et al., 2011).

Light quality and quantity remain one of most important challenges when experiments have to be carried out in growth chambers, since the plants will be exclusively dependent on artificial illumination that clearly differs from natural sun-light spectra. The aim of this study was to investigate the effects of different LEDs of continuous spectrum or FL light on the growth traits of *Q. ithaburensis* seedlings, during their indoor and nursery cultivation period. Our first hypothesis was that the effect of LEDs on early seedling’s growth would out-perform those of FL during indoor cultivation. Secondly that the beneficially growth traits initially induced by LED pre-treatments would still be detectable after a yearly nursery period that would eventually promote seedling’s quality. During indoor cultivation the evaluation of the different light qualities effects on *Q. ithaburensis* growth was tested by both morphological and physiological measurements. Nursery performance of the oak seedlings was based on morphological quality attributes that could be considered as a physical manifestation of their physiological activities, as well.

## Materials and Methods

### Plant Material

Acorns of *Q. ithaburensis* were collected from plantations placed in the Forest Research Institute located in Thermi, Vasilika, Thessaloniki, Greece (40°32′ 54.67′ N, 23°1′ 10.72′ E). Immediately after the collection, acorns were transferred to laboratory and immersed into water for 24 h. After 5 min, all of those still floating were removed. In order to succeed faster and more uniform germination of acorns their pericarp was totally peeled off, due to the presence of inhibitory substances that had been found in several oak species such as *Quercus nigra* (Peterson, 1983). Also by this sense prior to sowing the 1/3 of the distal ends of the acorns were cut off as suggested by Hou et al. (2010) and Giertych and Suszka (2011).

### Growth Chamber Characteristics and Conditions

The experiment of indoor cultivation phase was conducted into two environmentally control growth chambers at the Forest Research Institute in Greece (Hellenic Agricultural Organization-DEMETER). Both chambers reach 2 m height and consist of three shelves (1.20 m length, 0.60 m height, and 0.55 m depth; and the distance from the lights to the top of the plant material in the mini-plugs is 0.40 m). In chamber 1, the first shelf had four L20AP67 LED tubes, the middle had (of chamber one only) four fluorescent lamps (FL) [Osram, Fluora, Munich, Germany] as the reference lighting type, with 30 cm space between them and the bottom shelf had AP673L LED light. In chamber two the top shelf had G2 LED light, the second had AP67 LED light and the third shelf had NS1 LED light. LED light qualities were provided by Valoya LED lights (Valoya Oy, Helsinki, Finland) and the selected light spectrum percentages are shown in **Table 1**. A total of 48 acorns were sown in two different mini-plug plastic container trays (DL48 R – 315 × 550; cell size 45 × 53; depth 5 mm; volume 80 cm^3^; 273 plant/m^2^) (QuickPot by HerkuPlast-Kubern, Germany), containing enriched peat (Klassmann TS1, Klassmann-Deilmann GmbH, Geeste, Germany) mixed with perlite on the surface. Mini-plug trays were transferred to the environmentally controlled growth chambers for a cultivation period of 28 days. Thus in total 96 acorns were sown in each of the six shelves (each light spectrum) of the growth chambers. The environmental conditions inside the chambers consisted of 17 h photoperiod, 150 ± 10 μmol m^-2^ s^-1^ PPFD, 70 ± 10% air RH, and 20°C/15°C day/night temperature. Irradiance and PPFD of light sources were quantified at plant height with an LI-1800 portable spectroradiometer (LI-COR, Lincoln, NE, USA) with the sensor at ≈25 cm from the light tubes in 10 different spots through the growth chamber shelves. Watering was applied twice a day by automatic sprinklers at 9.00 a.m. and 24.00 a.m. for duration of 20 s; followed by full rotation of the trays in order to ensure uniform growth conditions.

**Table 1:** Spectral distribution and red:far-red ratio of the six light treatments.

Light treatments	Continuous spectrum data including different percentages out of 400–800 nm, %					R:FR ratio
	<400 nm	400–500 nm	500–600 nm	600–700 nm	700–800 nm	
FL	0	34.8	24.1	36.7	4.4	5.7
L20AP67	0	10.5	26.2	48.9	14.4	2.9
AP673L	0	11.9	19.3	60.5	8.3	5.6
G2	0	7.7	2.4	64.4	25.5	2.5
AP67	0	13.8	15.1	53	18.1	2.7
NS1	1.0	20.2	38.9	35.7	5.2	8.2

### Dry Weight

Dry weight matter accumulation of *Q. ithaburensis* seedlings showed significant differences among the light qualities only in the roots (**Table 2**). Thus seedlings under NS1 and AP673L LEDs had greater RDW of 3.18 and 2.9 g compared to the FL light that had 0.77 g (**Table 2**). The rest of LEDs such as AP67, G2, and L20AP67 showed also greater RDW than the FL but no significant differences were found (**Table 2**). LED lights also induced higher dry weight accumulation of leaves and shoots compared to FL; however, no significant differences were observed (**Table 2**).

**Table 2:** Leaf, shoot, root dry weight, (LDW, SDW, RDW) and root-to-shoot ratio (R/S) of *Quercus ithaburensis* seedlings cultivated under the FL, L20AP67, AP673L, G2, AP67, and NS1 light treatments at the end of the 28 days experimental period in the growth chambers.

Light treatments	LDW	SDW	RDW	R/S ratio
FL	0.25 ± 0.70a	0.29 ± 0.07a	0.77 ± 0.28b	0.96 ± 0.24bd
L20AP67	0.52 ± 0.15a	0.70 ± 0.09a	1.22 ± 0.55ab	1.37 ± 0.32bc
AP673L	0.52 ± 0.07a	0.65 ± 0.07a	2.9 ± 0.26a	2.40 ± 0.18c
G2	0.44 ± 0.09a	0.54 ± 0.11a	2.23 ± 0.53ab	2.30 ± 0.59c
AP67	0.52 ± 0.15a	0.58 ± 0.34a	2.26 ± 1.3ab	1.92 ± 0.28abcd
NS1	0.48 ± 0.12a	0.59 ± 0.08a	3.18 ± 0.29a	2.91 ± 0.58ac

*Different letters within the column indicate significant differences among the light treatments at *p* < 0.05. Data are mean values (*n* = 10) ± SE.*

### Experimental Design of Indoor Cultivation

From the total of the 96 seedlings in each shelf or light treatment 10 seedlings were randomly selected and marked. The six light spectra are the six levels of the treatment. Since seedlings are grouped into homogenous mini-plug trays, and 10 seedlings, were randomly selected per treatment, the experiment is defined as a randomized block design. In order to examine the averaging effect of replication that reflects the effectiveness of treatments, we repeated the measurements four times for the growth rate and leaf number (after 7, 14, 21, 28 days). At the end of 28 days, the randomly selected seedlings of the fourth replication were measured for morphological and physiological parameters.

### Measurements during the Indoor Cultivation Period

During the 28-days cultivation period, 10 seedlings per light treatment were randomly selected, marked and measured for their growth rate and leaf number four times. Growth rate based on the height increment was taken from each seedling every 7 days.

### Measurements at the End of the Indoor Cultivation Period

#### Stomatal and Epidermal Cells Assay

The impression approach was used to determine leaf SD, which was expressed as the number of stomata per unit leaf area (Radoglou and Jarvis, 1990). Mature leaves were used to produce impressions, prepared with clear nail polish parallel to the midrib, from the basal to apical position, on the abaxial surface, for approximately 20 min. The thin film (approximately 5 mm × 315 mm) was peeled off from the leaf surface, mounted on a glass slide, immediately covered with a cover slip, and then lightly pressured with finepoint tweezers. All the impressions were taken from at least five leaves (of five different seedlings) for each light treatment and examined under a light microscope with camera attachment (x40 magnification) using the Axio Vision program (47.1). Three fields per slide were randomly selected and photographed. Stomata and epidermal cells were counted on the photographs and the SD, SI, and CD were calculated. SI was estimated using the formula [s/(e + s)]x100 where s is the number of stomata and e is the number of epidermal cells (Salisbury, 1927). The guard cells were not included in the number of epidermal cells. CD was calculated as the total number of cells (e + s) per unit area of leaf.

#### Morphological and Physiological Parameters Assay

Leaf area was measured by the device LI-3000C Portable Area Meter (LI-COR Biosciences, Lincoln, NE, USA); CCI considered as the ratio between leaf transmission percentages at 931 nm and 653 nm was measured by the portable CCM-200 (Opti-Sciences, Inc., Hudson, New Hampshire, USA). The CCM sensor area is 0.71 cm^2^ and was placed randomly on the leaf mesophyll, avoiding the mid-vein. Five measurements were taken (per leaf) that were averaged to provide a single CCI per leaf. The saturation pulse method associated with the pulse-amplitude-modulation technique was applied for chlorophyll fluorescence measurements using a fluorometer MINI-PAM (Heinz Walz, Elleltrich, Germany). At the start of each of the three measurements held (on five different plants for each of the light treatments) plants were dark adapted for 20 min for determination of F_o_ and F_m_. The tip of the fiberoptics was located 1.0 cm from and 60° to the leaf surface. The effective quantum yield was calculated as ΔF/F_m_′ = (F_m_′ – F)/F_m_′, where F and F_m_′ are the fluorescence yield before and after the saturation pulse is applied on the leaf, respectively.

Also, selected seedlings were measured for the SH (cm) and the RL (cm) using a ruler. Before dry weight determination, the leaves (LDW), shoots (SDW), and roots (RDW) of the selected seedlings were oven-dried at 70°C for 48 h until a constant mass was reached. The dry weight was then measured using an electronic balance. In addition, the R/S was calculated on a dry weight basis.

#### Root Architecture Assay

For the root architecture analysis, five *Q. ithaburensis* seedlings per light treatment were selected, in order to separate their root section that was first gently washed out of the soil and subsequently scanned. Scanned photos of seedling’s root system were loaded in GiA Roots (Galkovskyi et al., 2012), which is a software tool to automate and facilitate the large-scale analysis of root networks. GiA Roots was used to quantify the structure of plant root system architecture by means of measuring the number of lateral roots; thus, estimation of the root density could be performed. Therefore, root density was calculated by computing the following formula: Root density = Length of primary root/no. of lateral roots (Bors-Oprisa et al., 2011). Additionally, root architecture of seedlings was also defined by the number of FOLRs greater than 1 mm diameter (primary FOLR) originating along the length of the taproot and at the base of the taproot. Thus, individual seedling root systems were assigned a fibrosity class on a 1–5 scale (five being the most fibrous) based on a root fibrosity index designed by Hatchell and Muse (1990) and modified by Wilson et al. (2007), to provide a relative measure of structural and fine root branching (**Table 3**).

**Table 3:** Rating system for root fibrosity of *Q. ithaburensis* cultivated under FL, L20AP67, AP673L, G2, AP67, and NS1 light treatments at the end of the 28 days experimental period in the growth chambers.

Light treatments	No of FOLR with d > 1 mm	Rating	Fibrosity class	Description of root system appearance
FL	1.4b	1b	Very low	No 2nd order long roots; zero or few short roots present
L20AP67	2.5b	3a	Moderate	3–5 2nd order long roots; moderate density of higher order long and short roots
AP673L	5ab	5a	Very high	5 > 5 2nd order long roots; high density of higher order long and short roots
G2	3.6ab	4a	High	>5 2nd order long roots; moderate density of higher order long and short roots
AP67	6.3a	>5a	Very high	5 > 5 2nd order long roots; high density of higher order long and short roots
NS1	6.6a	>5a	Very high	5 > 5 2nd order long roots; high density of higher order long and short roots

*Different letters within the column indicate significant differences among the light treatments at *p* < 0.05. Data are mean values (*n* = 5) ± SE. The rating is based on visual assessment of the approximate number and type of high order lateral roots per 10 cm segment of primary FOLRs (those with a diameter > 1 mm, branching from the taproot). Rating: (1) Very low (no second order long roots; zero or few short roots present); (2) Low (one–three second order long roots; low density of higher order long and short roots); (3) Moderate (three–five 2nd order long roots; moderate density of higher order long and short roots); (4) High (> five 2nd order long roots; moderate density of higher order long and short roots); (5) Very high (> five 2nd order long roots; high density of higher order long and short roots). Long roots are >5 mm and are likely to contain branches of the next highest order. Short roots are <5 mm; they do not support roots of higher order (Hatchell and Muse, 1990).*

### Nursery Characteristics and Conditions

Seedling quality attributes after their pre-cultivation under LEDs and FL light were investigated at Forest Nursery located at Nea Chalkidona, Greece (38.0275° N, 23.7308° E). The experiment was conducted in open-air (real) conditions. The seedlings were irrigated with an overhead irrigation system and no shading was used. The containers used were the Quick pots 24 T/16; these are plastic containers with 24 cavities each. Their cavity volume was 330 cm^3^ and the cavity depth was 16 cm. The soil substrate used was a mixture of peat and perlite (70:30, v/v), one of the most usual substrates in Greek nurseries. The peat was *Sphagnum* Lithuanian peat of medium structure and the perlite was of coarse structure. Also 5–10% of the total volume of loose clay sandy soil was added, enhanced with fertilizers such as 1.3 kg mixed fertilizer (N:P:K 15:30:15+micronutrients), 0.6 kg potassium sulfate, 1.0 kg super-phosphate (0–20–0), 0.4 kg magnesium sulfate and 2 kg lime (CaO) per m^3^ peat. *Q. ithaburensis* seedling’s growth was monitored for 1 year, while their quality assessment was based on the examination of physiological and morphological attributes, biannually.

### Experimental Design of Nursery Cultivation

Nursery experiment lasted for 1 year. The six light pre-treatments were arranged in a randomized complete block design with two replications. There were 24 seedlings per treatment per replication (total 288 seedlings). Every 6-month period 10 randomly selected seedlings per treatment (five seedlings × two replications) were collected for both non-destructive and destructive measurements.

### Nursery Measurements

At the end of the first nursery growth period (6 months in November), the seedling survival (%) was recorded for all light pre-treatments. Thereafter 10 randomly selected seedlings per light treatment were collected for non-destructive measurements such as leaf number, CCI, plant height (*h*), shoot height (*h*) (with an accuracy of 0.1 cm) shoot (*d*) (0.5 cm above root collar) and root diameter (*d*) (5 mm above the cotyledon scar), respectively. The same randomly selected seedlings were collected and transferred to the laboratory for destructive measurements such as biomass and seedling quality index estimations, while the remaining seedlings were kept in order to be measured at the following 6 months, thus at the end of 1 year growth. For biomass measurements, the seedlings were divided into three parts: leaves, shoots, and root system that were oven dried at 70°C for 48 h and then they were weighed. The R/S was calculated by the root and SDWs (Thompson, 1985). The seedling quality index (QI) was calculated using the equation (Dickson et al., 1960): DQI = total seedling dry weight (g)/[height(cm)/root collar diameter (mm)+shoot dry weight (g)/root dry weight (g)].

### Statistical Analysis

Statistical analysis was conducted with IBM SPSS Statistics for Windows, Version 20.0. Collected data every 7 days such as mean growth rate and leaf number under the different light treatments were analyzed using general linear model (GLM) Repeated Measurements. At the end of the 28th-day indoor cultivation period and both of the biannual nursery phases collected data from the randomly selected seedlings, were analyzed using GLM Multivariate Analysis. Significant differences were established by multiply comparisons test with Bonferroni correction at *p* < 0.05.

## Results

### Growth Rate

**FIGURE 1:**
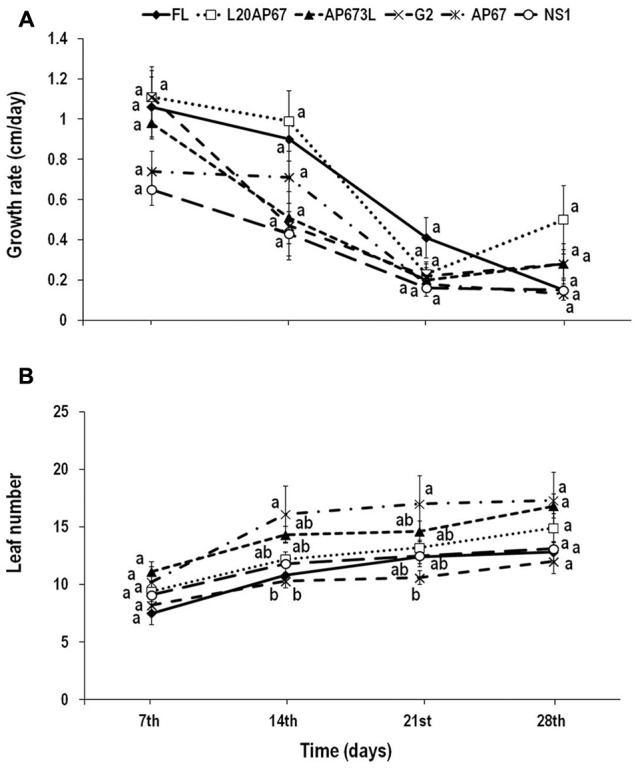
**Growth rate (height increment in cm every 7 days) (A)** and leaf number **(B)** of *Quercus ithaburensis* seedlings cultivated under the FL, L20AP67, AP673L, G2, AP67, and NS1 light treatments during a 28-day period. Different letters within the lines indicate significant differences among light treatments at *p* < 0.05. Data are mean values (*n* = 10) ± SE.

*Quercus ithaburensis* seedlings showed no significant differences among the different light treatments for the height growth rate over time. Hence, all the light treatments presented a similar height increment for each measuring date of the indoor experiment (**Figure 1A**). However, considering only the light effect on the height increment, it was higher for the L20AP67 of 0.71 cm, followed by the FL, G2, AP673L, AP67, and NS1 of 0.63, 0.52, 0.50, 0.44, and 0.35 cm, respectively. Time effect was also significant, showing a general reduction of the height increment for all the light treatments until the 21st day into the growth chambers.

### Leaf Number

Significant differences among the light treatments and over time were found at the 14th and 21st day of the indoor experiment. AP67 LED light showed significantly faster leaf formation of 43.93 and 39.4% compared to FL and G2 lights (**Figure 1B**) during the 14th day. Also at the 21st day AP67 LED showed significantly faster leaf formation of 42.85% compared to the G2 LED (**Figure 1B**). At the end of the experiment, seedlings under AP67 LED light induced the highest number of leaves, however, no significant differences were found among the light treatments, while those under both G2 and FL had the least.

### Stomatal Density (SD) – Stomatal Index (SI) (%) – Cell Density (CD)

**FIGURE 2:**
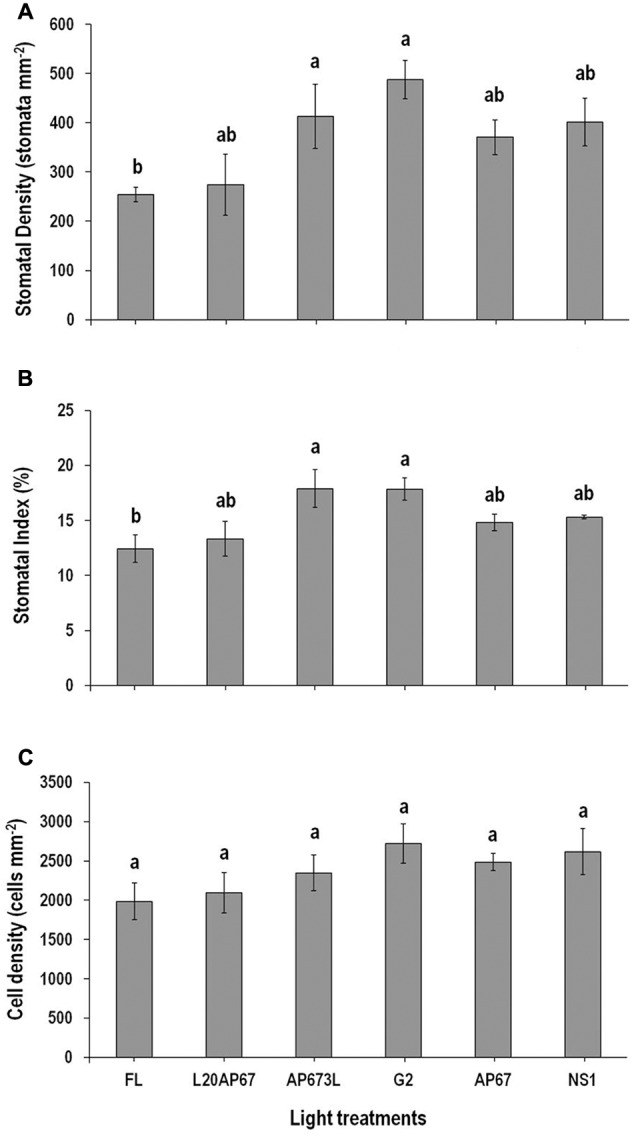
**Stomatal density (SD) (A)**, stomatal index (SI) (%) **(B)**, and cell density (CD) **(C)** on the abaxial leaf surface of *Q. ithaburensis* seedlings cultivated under the FL, L20AP67, AP673L, G2, AP67, and NS1 light treatments at the end of the 28 days experimental period in the growth chambers. Different letters within the columns indicate significant differences among the light treatments at *p* < 0.05. Data are mean values (*n* = 5) ± SE.

Stomatal density was significantly higher of 488 stomata/mm^2^ and 412.6 stomata/mm^2^ on leaves grown in the presence of G2 and AP673L LEDs compared to the FL with SD of 254.3 stomata/mm^2^ (**Figure 2A**). Seedlings under the rest of the light treatments such as NS1, AP67, and L20AP67 also showed higher SD than the FL, but no significant differences were found (**Figure 2A**). G2 and AP673L LEDs induced also significantly higher SI of 17.8% than the FL with SI of 12.4% (**Figure 2B**). As for the rest of the LEDs no significant differences were found (**Figure 2B**). Different light qualities showed no significant differences for the CD; however CD was higher under LED lights than the FL (**Figure 2C**).

### Leaf Area, Chlorophyll Content Index, and Chlorophyll Fluorescence

None of the specific parameters measured on the oak seedlings, showed significant differences irrespective the different light qualities used. Leaf area of the seedlings under all lights was similar of 84.4 cm^2^ (data not shown). The CCI was 16.5 similar under all light treatments, while F_v_/F_m_ was close to 0.8 for all lights indicating no photochemical damage of PSII system for the oak seedlings (data not shown).

### Morphological Parameters

#### Shoot Height (SH) and Root Length (RL)

**FIGURE 3:**
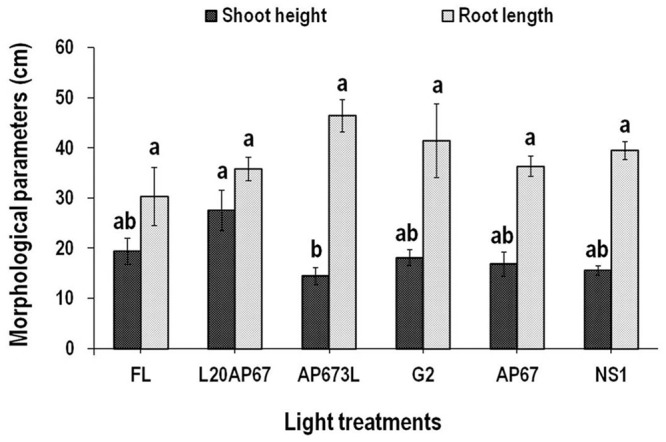
**Shoot height (SH) and root length (RL) of *Q. ithaburensis* seedlings cultivated under the FL, L20AP67, AP673L, G2, AP67, and NS1 light treatments at the end of the 28 days experimental period in the growth chambers.** Different letters within the columns indicate significant differences among the light treatments at *p* < 0.05. Data are mean values (*n* = 10) ± SE.

L20AP67 LED light induced significantly higher SH of 27.5 cm for the oak seedlings compared to those of AP673L LED light that were more compact (**Figure 3**), while no significant differences were found for the rest of the light treatments (**Figure 3**). The RL of the *Q. ithaburensis* seedlings was unaffected by the different light qualities, however, those under the AP673L LED light had longer roots (**Figure 3**).

#### Root-to-shoot Ratio (R/S)

Significantly higher R/S ratio of 2.9 by means of higher dry weight matter allocation to the seedlings roots was found under the NS1 LED, compared to the FL and L20AP67 that had R/S ratio of 0.96 and 1.37, respectively (**Table 2**). Furthermore, both AP673L and G2 LEDs induced significantly higher R/S ratio of 2.4 and 2.3 than the FL (**Table 2**).

### Root Architecture

#### Root Density and Root Fibrosity

Root density of *Q. ithaburensis* seedlings was higher under LED lights than the FL light by means of greater number of FOLRs; however, no significant differences were found (data not shown). Nonetheless oak seedlings under NS1 and AP67 LEDs had significantly higher number of FOLRs with diameter greater than 1 mm of 6.6 and 6.3 compared to FL and L20AP67 that had 1.4 and 2.5 (**Table 3**). As for the rest of LEDs such as AP673L and G2 induced also higher number of 5 and 3.6 than the latter lights mentioned; however, no significant differences were found. Thus, seedlings grown under all LED lights formed a root system of high density of the higher order lateral roots than the FL light as shown in **Table 3**.

### Nursery Performance

#### Seedling Survival (%)

**FIGURE 4:**
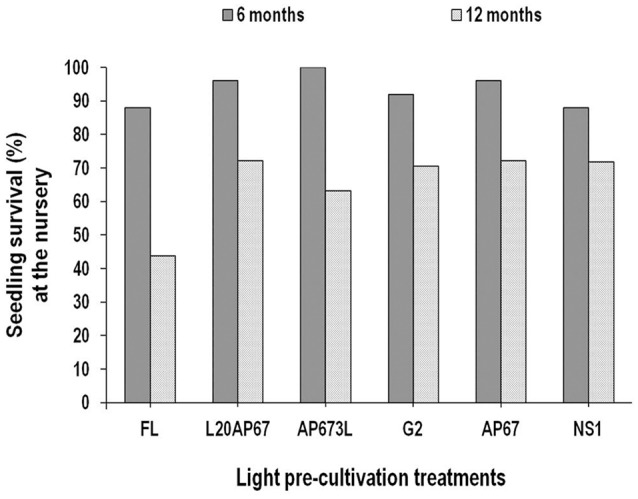
**Seedling survival (%) after six and twelve months at nursery of *Q. ithaburensis* seedlings pre-cultivated under the FL, L20AP67, AP673L, G2, AP67, and NS1 light treatments**.

After 6 months at the nursery high seedling survival was succeeded for all light pre-treatments (**Figure 4**). Especially for the AP673L LED light that shown 100% survival, followed by those of AP67 with 96% seedling survival, L20AP67 of 95%, G2 of 92%, NS1 and FL of 87%. At the end of the 12-month period at the nursery, seedling survival of *Q. ithaburensis* showed a general decrease for all lights; however, the highest was for the FL (-56.2%), followed by the G2 (-29.4%), NS1 (-28.1%), L20AP67 (-27.7%), AP67 (-27.7%), and the lowest for AP673L (-26.3%).

#### Number of Leaves

After a 6-month period that *Q. ithaburensis* seedlings grown in the nursery, no significant differences were found for the leaf number among the different light qualities; however more leaves were formed for the AP673L LED (data not shown). At the end of 12 months at the nursery, the number of leaves of all the light treatments showed up to 55% significant increase; however, no significant differences were found among them, while more leaves were formed for the AP67 LED pre-cultivated seedlings and lower for those of FL (data not shown).

#### Chlorophyll Content Index (CCI)

**FIGURE 5:**
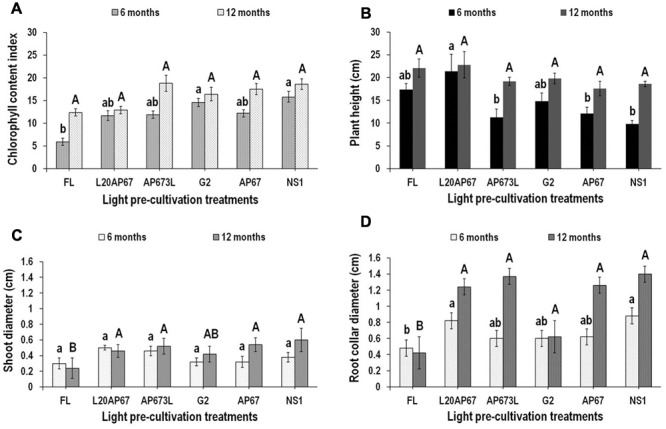
**Chlorophyll content index (CCI) (A)**, plant height **(B)**, shoot diameter (ShD) **(C)**, and root collar diameter (RCD) **(D)** after six and twelve months at nursery of *Q. ithaburensis* seedlings pre-cultivated under the FL, L20AP67, AP673L, G2, AP67, and NS1 light treatments. Different lowercase letters within a column indicate significant differences among light treatments at the end of six months while uppercase letters indicate significant differences among light treatments at the end of 12 months at *p* < 0.05. Data are mean values (*n* = 10) ± SE.

Seedlings that were pre-cultivated under NS1 and G2 LEDs showed significantly higher CCI values of 15.8 and 14.6 compared to FL light that had 5.9 at the end of 6 months at the nursery (**Figure 5A**). However, between the two-time intervals that our measurements were held, FL, AP673L and AP67 lights marked significant increase of 71, 45, and 35% in CCI, respectively. Nonetheless at the end of 12 months at the nursery no significant differences were found among the lights for the CCI, however, it was higher for the LED pre-treatments than FL (**Figure 5A**).

#### Plant Height

At the end of the 6-month nursery period, seedlings of L20AP67 had significantly greater plant height of 21.4 cm compared to those of NS1, AP673L, and AP67 that were 9.8, 11.3, and 12.1 cm high, respectively (**Figure 5B**). At the end of the12 months at the nursery oak seedlings did not show significant differences for their plant height among the light pre-treatments; however, seedlings of NS1, AP673L, AP67, and G2 showed significant increase of 62, 51.8, 37, and 33% in their plant height between the two-time intervals, while seedlings of FL and L20AP67 had a non-significant increase of 23.8 and 6.3%, respectively.

#### Shoot Height (SH)

No significant differences were found among the light pre-treatments for the SH of the oak seedlings either at the end of the 6- or 12-month period at the nursery (data not shown). However significant SH increase was found for the seedlings of NS1, AP673L, AP67, and G2 between the two-time intervals of 66.1, 52.5, 38.5, and 34.4%, respectively, while FL and L20AP67 lights had an increase of 17.4 and 25.5%, but was not significant.

#### Shoot Diameter (ShD)

No significant differences were found among the lights for the ShD after 6 months at the nursery (**Figure 5C**). However, seedlings of AP67 and NS1 LEDs had a significant ShD increase of 51 and 44.8% between the two time intervals. At the end of the 12-month nursery period seedlings of NS1, AP67, AP63L, and L20AP67 had significantly higher ShD of 0.60, 0.54, 0.52, and 0.56 cm compared to those of FL that had ShD of 0.24 cm (**Figure 5C**).

#### Root Collar Diameter (RCD)

After 6 months at the nursery, *Q. ithaburensis* seedlings of NS1 and L20AP67 LED pre-treatments had significantly higher RCD of 0.88 and 0.82 cm compared to those of FL with RCD of 0.44 cm (**Figure 5D**). At the end of the 12-month nursery period the seedlings of all LED pre-treatments had significantly higher RCD compared to those of FL light (**Figure 5D**). Furthermore, seedlings of G2 LED pre-treatment had significantly lower RCD compared to the rest of LEDs (**Figure 5D**). Also between the two time intervals the seedlings that pre-cultivated under AP673L, AP67, NS1, and L20AP67 LEDs showed significant increase of 78.2, 68, 47.6, and 40.7% in their RCD.

#### Dry Weight

After 6 months of growth at the nursery *Q. ithaburensis* seedlings of AP673L LED pre-treatment had significantly higher LDW compared to those of FL (**Table 4**), while the seedlings of the rest of the LED lights such as AP67, L20AP67, NS1, and G2 had also higher LDW than FL of 0.550, 0.524, 0.522, and 0.452 g, but no significant differences were found. No significant differences were also not found for the SDW among the light pre-treatments, however it was higher for the LEDs (**Table 4**). The seedlings of all LEDs had significantly higher RDW compared to those of FL (**Table 4**). In addition, NS1 LED pre-cultivated seedlings had significantly higher RDW than those of AP673L (**Table 4**).

**Table 4:** Leaf, shoot, root dry weight, (LDW, SDW, RDW) and root-to-shoot ratio (R/S) after six and twelve months at nursery of *Q. ithaburensis* seedlings pre-cultivated under the FL, L20AP67, AP673L, G2, AP67, and NS1 light treatments.

Light treatments	6 months at nursery				12 months at nursery			
	LDW	SDW	RDW	R/S ratio	LDW	SDW	RDW	R/S ratio
FL	0.35 ± 0.04b	0.70 ± 0.05a	2.26 ± 0.72b	2.16 ± 0.70b	0.28 ± 0.08B	0.31 ± 0.09B	1.13 ± 0.55B	1.95 ± 1.10B
L20AP67	0.52 ± 0.04ab	1.01 ± 0.16a	5.37 ± 0.09acd	3.54 ± 0.37abd	0.57 ± 0.18A	0.76 ± 0.13AD	5.43 ± 0.33A	4.19 ± 0.97A
AP673L	0.62 ± 0.10a	0.81 ± 0.03a	5.00 ± 1.48ad	3.45 ± 0.80abd	0.62 ± 0.14A	0.84 ± 0.15AD	5.05 ± 0.48A	3.51 ± 0.81AB
G2	0.45 ± 0.04ab	0.81 ± 0.17a	5.66 ± 0.66acd	4.48 ± 0.56acd	0.52 ± 0.17AB	0.81 ± 0.23AD	4.29 ± 1.21A	2.23 ± 0.35AB
AP67	0.55 ± 0.02ab	0.82 ± 0.20a	6.69 ± 1.34acd	4.87 ± 0.71acd	0.67 ± 0.22A	1.23 ± 0.41AC	5.80 ± 0.50A	3.28 ± 1.06AB
NS1	0.52 ± 0.08ab	0.79 ± 0.25a	5.59 ± 1.06ac	5.37 ± 0.80ac	0.49 ± 0.14AB	0.86 ± 0.11ACD	5.59 ± 1.17A	4.18 ± 1.11A

*Different lowercase letters within a column indicate significant differences among light treatments at the end of six months while uppercase letters indicate significant differences among light treatments at the end of 12 months at *p* < 0.05. Data are mean values (*n* = 10) ± SE.*

After 12 months at the nursery seedlings of AP67, AP673L, and L20AP67 LED pre-treatments had significantly higher LDW compared to those of FL (**Table 4**). Also, significantly higher SDW had the seedlings of all LED pre-treatments compared to the FL (**Table 4**). In addition, seedlings of AP67 LED pre-treatment had significantly higher SDW compared to those of L20AP67, G2, and AP673L (**Table 4**). FL seedlings had the lowest RDW and significant differences were found with all LED pre-treatments (**Table 4**).

In total LDW of the *Q. ithaburensis* seedlings showed no significant increase between the two-time intervals, irrespective the light spectrum of their previously indoor pre-cultivation. In contrast, significant decrease of 75.8% for SDW was induced for the FL seedlings and significant increase of 40.2% for the AP67 seedlings. Furthermore, significant decrease of 27.5 and 20.7% for the RDW of G2 and NS1 LEDs was observed.

#### Root-to-shoot Ratio (R/S)

Significantly higher allocation to the roots than to the above parts of the plants after 6 months at the nursery was found for the pre-cultivated seedlings of NS1, AP67, and G2 with R/S ratio of 5.3, 4.8, and 4.4 compared to those of FL with ratio of 2.1, which was the lowest. In addition, NS1 LED pre-cultivated seedlings had significantly higher R/S ratio than those of L20AP67 and AP673L (**Table 4**). After 12 months at the nursery, L20AP67 and NS1 LED pre-cultivated seedlings had significantly higher R/S ratio of 4.1 compared to those of FL that had 1.9 (**Table 4**), while seedlings of the rest of the pre-treatments such as AP673L, AP67 and G2 showed also higher R/S ratio than the latter light, but no significant differences were found. Moreover, significant decrease of 39, 32.4, and 24.9% for the R/S ratio of AP67, G2 and NS1 pre-cultivated seedlings was found between the two time intervals.

#### Dickson’s Quality Index (DQI)

**FIGURE 6:**
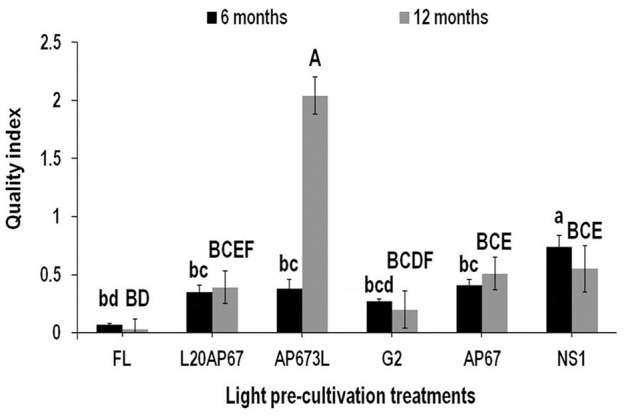
**Dickson’s quality index (DQI) after six and twelve months at nursery of *Q. ithaburensis* seedlings pre-cultivated under the FL, L20AP67, AP673L, G2, AP67, and NS1 light treatments.** Different lowercase letters within a column indicate significant differences among light treatments at the end of six months while uppercase letters indicate significant differences among light treatments at the end of 12 months at *p* < 0.05. Data are mean values (*n* = 10) ± SE.

Dickson’s quality index was a reliable predictor of seedling field performance for those pre-cultivated under LEDs than the FL light. After 6 months at the nursery seedlings of NS1, AP67, AP673L, and L20AP67 had DQI of 0.74, 0.41, 0.38, and 0.35 that differed significantly with the DQI of 0.07 of FL seedlings (**Figure 6**). In addition, NS1 LED pre-cultivated seedlings had significantly higher DQI compared to all LED pre-treatments (**Figure 6**). After 12 months FL pre-cultivated seedlings still had significantly lower DQI of 0.03 compared to those of AP673L, NS1, AP67, and L20AP67 (**Figure 6**). Furthermore, AP673L LED pre-cultivated seedlings had the highest DQI and highly significant differences were found with all LEDs (**Figure 6**). Also NS1 and AP67 LED pre-cultivated seedlings had significantly greater DQI than those of G2 (**Figure 6**). Highly significant increase of 137% of the DQI was found for the AP673L seedlings between the two time intervals, while the DQI of NS1 seedlings showed significant decrease of 29.4%.

## Discussion

*Quercus ithaburensis* seedlings showed similar and slow growth rate, irrespective of the different light quality irradiations, during the whole indoor experimental period. Those findings may partially be explained by the fact that *Q. ithaburensis* is a large-seeded species and according to the so-called “metabolic effect,” species with larger seeds have a slower relative growth rate especially during the early growth stages, due to the dependence on endogenous factors (Baraloto et al., 2005; Bentsinka and Koornneef, 2008; Montagnoli et al., 2016). Besides that, *Q. ithaburensis*, even though it is considered as a xero-thermic species with a high light demand, its young seedlings may tolerate medium levels of side shadowing (Huxley, 1992). Many plants are sensitive to changes in the red:far-red light ratio (R:FR), which can be considered, depending on plant species and/or light treatment, as a signal of shading and proximity to other plants (Gilbert et al., 1995; Page et al., 2010). Therefore, usually plants respond to this factor by modifying certain morphological and physiological traits as mechanisms of shade avoidance (Tegelberg et al., 2004), such as enhanced elongation, a strategy employed by the majority of angiosperms, ranging from small herbs to large trees (Casal, 2012). However, our findings revealed only a tendency of faster height increment through time for the seedlings under L20AP67 LED that had a low R:FR ratio (2.9) and slower for those grown under NS1 that had the highest R:FR ratio of 8.1 among all the light treatments. According to Bantis et al. (2016), both *Ocimum basilicum* cultivars (Lettuce Leaf, and Red Rubin-mountain Athos hybrid), that were tested under the same light treatments as in our study, had significantly higher growth rate under G2 and AP67 LEDs (with low R:FR ratios of 2.5 and 2.7) compared to the lowest obtained under NS1 and AP673L LEDs, therefore, those different responses on height growth rate may indicate species dependence.

*Quercus ithaburensis* seedlings had significantly greater leaf number under AP67 LED in the midst of the indoor experiment (14th and 21st week), compared to G2 and FL lights. Actually, faster and higher leaf formation of the seedlings during the whole experimental period was found under AP67 and AP673L lights, while the exact opposite was found for the G2 and FL lights. Although, according to Lee et al. (2015), the leaf number of red lettuce (*Lactuca sativa* ‘Sunmang’), was significantly higher in most R:FR treatments than the FL lamps, especially for the 1.2 and 8.6 ratios, while the 4.1 R:FR ratio had the lowest. Also the emergence and leaf expansion of zygotic embryos and *in vitro* plantlets of open-pollinated chestnut (*Castanea crenata* S. et Z) was faster under blue than under red monochromatic LED radiation (Park and Kim, 2010). In our case AP67 LED had a low R:FR of 2.7, while G2 had the lowest R:FR of 2.5 and the highest percentages of 64.4 and 25.5% in the red and far-red area, respectively among all lights. On the other hand, AP673L had a high R:FR ratio of 5.5 and FL even higher R:FR of 5.7. Therefore, the amount of red and far-red light absorbed could determine the phytochrome status of a plant due to the inter-conversion between a biologically inactive (P_R_) and an active (P_FR_) form, thus irradiation with more far-red light results in shade avoidance syndrome (SAS) (Casal, 2012), by affecting various responses such as stem elongation and leaf growth (Stutte, 2009; Hogewoning et al., 2010).

Plants must constantly adapt to the environmental variations, by regulating the gas exchange through stomata, thus allowing the optimization and balance of the photosynthetic performance (Chaerle et al., 2005) or modulate the frequency of stomata development in new organs (Lake et al., 2001). Nevertheless stomatal opening and density, is thought to be induced by distinct mechanisms of various photoreceptors depending on the wavelength of incident light (Shimazaki et al., 2007). In our case G2 and AP673L LED lights triggered significantly higher stomata number on the abaxial leaf surface of *Q. ithaburensis* seedlings, compared to those grown under FL and L20AP67 beside the fact that the latter LED light mentioned (L20AP67) showed a tendency of faster growth rate and greater leaf area. In contrast, epidermal cell number or CD was not affected by the different light qualities however, both were higher under all LEDs, especially under G2; may due to higher PAR levels derived from the unique continuous spectrum of each of the LED lights used than the FL light. In addition, there is no correlation between photosynthetic rate and SI in poplar (*Populus* spp.; Miyazawa et al., 2006) and tobacco (*Nicotiana tabacum*) plants, and apart from the reduced photosynthesis, normal responses of SD and SI to PAR (photosynthetically active radiation) was found, suggesting that other photoreceptors could be involved in this regulation (Baroli et al., 2008). Thus in our study the faster height increment did not imply necessarily greater stomatal development or frequency in oak seedlings.

The role of photoreceptors that mediate specific light signaling by promoting stomatal development during the early developmental stage of seedlings has not been revealed (Kang et al., 2009), even more so concerning a continuous light spectrum. Therefore, both G2 and AP673L LEDs had showed significantly higher SD and SI (%) compared to FL light. Both of LED light qualities had lower levels of blue radiation in their certain continuous spectrum or lower B:R ratios (of 0.12 and 0.19 respectively), among all light treatments, especially the G2 that had the lowest of all. Wada et al. (2003) emphasized the role of blue light on chloroplast development, chlorophyll formation and stomata opening. Lu et al. (1993) reported more than a threefold increase in upper SD when the blue/red ratio was increased from 0.81 to 1.08 in cotton leaves (*Gossypium barbadense* L.). In contrast, Rajapakse and Kelly (1993) reported a 10% decrease in SD in *Chrysanthemum* [*Dendranthema × grandiflorum* (Ramat.) Kitamura] when the blue/red ratio increased. Leptosporangiopsida ferns lack a blue light-specific stomata opening response, whereas the stomata open by red light (Doi et al., 2006). Green-light exposure reversibly decreases stomatal conductance in lettuce (Kim H.H. et al., 2004), while the combination of green light with blue and red light improves plant biomass and chlorophyll content in lettuce seedlings (Dougher and Bugbee, 2001). Therefore, in our case those responses may be triggered due to higher red radiation obtained or a possible synergistic effect between red and far-red light on stomatal development and indicates the suppression of the phototropins blue-mediated function at least for the G2 LED, while in the case of AP673L LED those responses may be brought on by green light radiation in concert with the specific blue and red radiation percentages.

Leaf area of *Q. ithaburensis* seedlings showed no significant differences irrespective the light spectrum. Later work on chrysanthemum (*Chrysanthemum morifolium*) has shown that blue light inhibited stem elongation, increased pigment content, decreased leaf area, and lowered total dry weight (Oyaert et al., 1999). LA was higher for the seedlings of L20AP67 light quality, which had 10.5% in blue radiation and low R:FR ratio of 2.9, while it was lower for NS1 that had twofold higher proportion in blue radiation (20.2%) and the highest R:FR ratio of 8.1, which means that the shade avoidance response is less prominent. Additionally, L20AP67 oak seedlings had significantly higher SH, greater participation in LDW and SDW among lights and significantly lower R/S ratio than those of NS1. Recent studies have reported that increased blue LED ratio increased leaf biomass, decreased leaf area and shoot biomass, developed sun adapted leaves, and had no effect on flowering in roses (Terfa et al., 2012). Thus, it could be assumed that the different responses caused by the varied proportions of blue radiation, are species dependent. Moreover, different light irradiations did not induce significant changes either in the CCI or the F_v_/F_m_ ratio that actually is close to 0.8, a value typical for uninhibited plants with a well-functioning photosynthetic apparatus (Björkman and Demmig, 1987).

Plants that can detect a low R:FR ratio will initiate a series of physiological changes and consequently express shade avoidance characteristics such as increased stem elongation, reduced stem diameter, and decreased root biomass (Page et al., 2010; Afifi and Swanton, 2011). In our study, the SH of L20AP67 seedlings was significantly greater than those of AP673L, which it was in agreement with our previous findings about the higher height growth rate maybe due to its low R:FR ratio. Further, higher tendency of promoted root development was found under AP673L may due to its high percentage in red radiation, which could induce leaf expansion and root development (Park and Kim, 2010; Johkan et al., 2012).

Combined red and blue LED lights were proven to be an effective lighting source for producing many plant species, in controlled environments (Dougher and Bugbee, 2004; Shin et al., 2008), while the dry weight in several species such as chrysanthemum, tomato and cucumber was promoted (Kim S.J. et al., 2004; Liu et al., 2011; Savvides et al., 2012). In this study *Q. ithaburensis* seedlings dry weight accumulation was far better predicted under the continuous spectrum of LED lights compared to FL light. In specific, both NS1 and AP673L LEDs induced almost fourfold increase for the RDW compared to FL light. Further, LDW and SDW also were greater under LEDs than the FL light. The significant increase of the RDW accumulation induced by the NS1 and AP673L LEDs may be due to the high percentages in green region accompanied by the lowest percentages in far-red radiation in their continuous spectrum, as well. As previously mentioned NS1 LED light had a low percentage of 1% covering the UV area (<400 nm), while its existence showed not an unfavorable effect concerning the RDW accumulation. Previous studies have reported that green light has unfavorable effects on plants, including decreased chlorophyll content, inhibited stomatal opening (Son et al., 2012), or growth inhibition (Terashima et al., 2009). However, green light is efficiently absorbed and used for photosynthesis in inner canopy levels (Folta and Maruhnich, 2007) and thus may stimulate growth in different plants and affect the morphogenetic processes through phytochrome and cryptochrome activity (Kim S.J. et al., 2004; Kim et al., 2006). In a far-red radiation regime reported by De la Rosa et al. (1998), Scots pine seedlings (*Pinus sylvestris* L.) showed increase in the SH and decrease in the total dry weight of seedlings, in specific the dry weight allocation to needles was not affected, whereas dry weight allocation to roots was reduced indicating a non-balance growth which could negatively affected tree performance over time.

In addition, Cen and Bornman (1990) found that bean plants (*Phaseolus vulgaris* L.) cultivated under low PAR regimes were affected by UV-B light, as shown by a stronger reduction in LDW. However, in our study oak seedlings were unaffected by the small percentage in UV region emitted from NS1 LED light which did not induce any decrease in the dry weight accumulation at least in the roots; actually, it induced the highest by far.

Seedlings grown in low blue light environments or enriched in the green region of the light spectrum also commonly exhibit shade avoidance characteristics such as increased height growth, and typically exhibit a reduced investment in organs such as roots or leaf blades (Zhang et al., 2011; Pierik and de Wit, 2014). However, in our case NS1 LED light is high both in blue and green region, actually it has the highest percentage of 38.9% in green (500–600 nm) among all lights, nevertheless induced the highest carbon allocation in the roots of *Q. ithaburensis* seedlings, by means of significantly higher R/S ratio compared to FL and L20AP67 lights; An increase in R/S ratio could be an indication of a healthier plant, provided the increase came from greater root size and not from a decrease in shoot weight, and expected to survive quite well under harsh environmental conditions (Lloret et al., 1999; Jacobs et al., 2009). Thus, AP673L and G2 LED lights that induced significantly higher SD which in turn could amplify the potential of greater carbon assimilation, also induced significantly higher R/S ratio of the seedlings than the FL, while had greater dry weight of leaves and shoots than the FL light, may due to their high content of red radiation or as a result of synergistic action of red and blue light, because it enhanced photosynthetic rates and showed greater gains in biomass (Poudel et al., 2008; Wu and Lin, 2012).

Root architecture of oak seedlings has been the subject of previous studies (Tamasi et al., 2005; Collet et al., 2006; Wilson et al., 2007). Actually, root architectural analysis allows a formal description of root systems and has important ecological applications since it reflects root plasticity responses to environmental heterogeneity and edaphic constraints to plant productivity and determines the function of roots in mechanical support of the shoots (McPhee, 2005; Sardans and Peñuelas, 2013). The development of a deep root system at the expense of shoot has been regarded as a crucial morphological adaptation of several oak species such as *Quercus suber* L., *Quercus petraea* Matt., *Quercus ilex* L., to compensate seasonal drought events in Mediterranean region (Ja et al., 2011; Kuster et al., 2013; Comas et al., 2015; Turcsán et al., 2016). Thus the initial growth and development strategy of oak seedlings could define the morphological structure of their mature root and shoot system (Tsakaldimi et al., 2013). Further, it is known that roots show various photoresponses, and light influences many aspects of root development including root extension, geosensitivity and lateral root formation (Poudel et al., 2008). Thus in our study, densely root systems (root density) were formed by means of greater number of lateral roots occupied per cm of the primary RL and greater number of FOLRs of *Q. ithaburensis* seedlings grown under LED lights than the FL. The majority of the photoreceptors and light signaling modules are expressed in roots (Warnasooriya and Montgomery, 2011); ultraviolet light of type-B controls many aspects of plant development, including root growth (Tanaka et al., 2002) or blue light has been observed to be involved in lateral root formation (Meng et al., 2015). Root fibrosity is a relative index of root branching thus in our study NS1 and AP67 LED light treatments modified the root system fibrosity of *Q. ithaburensis* seedlings by inducing significantly greater number of FOLRs with diameter >1 mm compared to FL conventional light and L20AP67 LED. Therefore, that attribute might enhance the potential to improve seedling quality even after out-planting, by means of greater growth and survival. Thus both LEDs showed enhanced dry weight allocation in the root system of oak seedlings.

Seedling survival after 6 months was high for all treatments especially for the AP673L that all seedlings survived (100% success). After 12 months at the nursery a general reduction of seedling survival was found for all treatments, however, it was highest (-56.2%) for the FL seedlings, while LED pre-cultivated seedlings still had high survival. Concerning the seedlings of oak species, the large number of primary FOLRs and the high root system fibrosity (root system with a relatively high root surface area and with a large number of root apices) are considered parameters that improve field survival and early growth of seedlings (Wilson et al., 2007). Indeed LEDs of continuous spectrum pre-cultivation as previously mentioned induced higher number of FOLRs and higher root fibrosity in the tested species. Moreover most seedling mortality occurs in the first dry season of their life cycle and it has been attributed, in addition to other factors, to poor stock quality (Gazal and Kubiske, 2004), thus the higher LED pre-cultivated seedling survival of *Q. ithaburensis* may be an indication of enhanced quality characteristics.

During the indoor cultivation phase, AP67 and AP673L LEDs were the light qualities that induced a tendency of faster leaf formation of *Q. ithaburensis* seedlings. Thus the more a plant invests in leaf expansion, the higher the total carbon gain and the faster growth will occur (Poorter and Remkes, 1990). Surprisingly the same higher tendency of greater leaf number still existed for the seedlings of AP67 and AP673L LEDs, after 6 months and even after 12 months at the nursery under natural light conditions.

Hogewoning et al. (2010) reported the important role of blue light in the chlorophyll biosynthesis; however, the CCI of *Q. ithaburensis* seedlings showed no significant differences irrespective of the light spectrum, during the indoor cultivation. That was not the case when the seedlings were for 6 months at the nursery under natural light. LED pre-cultivated seedlings had higher CCI, especially those of NS1 and G2, that beside their continuous spectrum had also the highest and the lowest R:FR ratio, respectively, were significantly higher compared to FL. In addition, the CCI was higher even after 12 months at the nursery for the LED pre-cultivated seedlings compared to FL, as well. Actually, the CCI of AP673L seedlings was the lowest during the indoor cultivation, eventually showed a significant increase of 45% between the two time intervals at the nursery and kept the highest of all treatments, which eventually led after 12 months to the high nursery performance of the seedlings by means of the highly significant increase of the DQI. In addition, AP673L LED induced significantly higher SD during the indoor cultivation, which it could amplify the potential of greater carbon assimilation, as well. Chlorophyll content is an important factor for photosynthesis, growth, and development of plants (Calatayud and Barreno, 2004), thus the higher CCI of the LED pre-cultivated seedlings was an indication of better adaptation under natural light conditions that would further lead to higher carbon assimilation rate per unit area.

Oak seedlings of L20AP67 LED treatment had significantly higher plant height compared to those of NS1, AP673L, and AP67 after 6 months at the nursery and had greater SH as well, which is also in agreement with the indoor cultivation findings where that light induced significantly taller seedlings, while NS1 and AP673L induced more compact seedlings. Even after 12 months the L20AP67 seedlings still kept those morphological traits. Taller seedlings may have a competitive advantage on sites with severe weed competition (Johnson and Smith, 2005), on the other hand taller seedlings with greater transpirational area may have a disadvantage on dry sites (McTague and Tinus, 1996); and exceptionally tall seedlings may be difficult to plant, out of balance (poor shoot-to-root ratio) and subject to wind damage (Speck, 2003). However, the greater plant height of the oak seedlings induced by the L20AP67 had not shown a non-balanced carbon allocation either to the above ground or the below ground parts of the plants, by means of high ShD, RCD, R/S ratio, and DQI (%) detected during the nursery growth phase.

Numerous studies showed larger shoot diameter seedlings tend to survive better than small shoot diameter seedlings (Mexal et al., 2008; Oliet et al., 2009; Morrissey et al., 2010). Also shoot diameter is closely related with root morphological traits particularly number of FOLRs (Corpuz, 2012). While it is possible that large diameter seedlings inherently have a more fibrous root system (Carandang, 1994). Seedlings of *Q. ithaburensis* showed similar values for the ShD for all pre-cultivation lights, after 6 months at the nursery. After 12 months, however, LED lights such as NS1, AP67, AP673L, and L20AP67 had significantly greater ShD than the FL. In conjunction with those findings, and the greater number of FOLR that previously had been found for all LEDs, indeed the latter statement is supported. Root-collar diameter has been recognized in previous studies as an important initial attribute of nursery seedlings to promote field survival particularly under drought conditions (South et al., 2001) because they are likely to have stored more carbohydrates and nutrients (Jacobs et al., 2009). Thus, our study revealed that the RCD of NS1 and L20AP67 LED pre-cultivated seedlings was significantly larger, than those of the FL after 6 months. Moreover, significantly larger RCD that was further enhanced after 12 months was found for the seedlings of all LEDs, except from those of G2 that was still higher than FL.

Pre-cultivation under different light treatments also had a significant effect on dry weight accumulation of seedlings that continued to be observed during the 6-month period at the nursery. Even more after 12 months, oak seedlings of all LED treatments, especially those of AP67 and NS1 had highly significant differences in the dry weight accumulation compared to those of FL. In addition, the R/S ratios of the seedlings that pre-cultivated under LEDs were significantly higher during the whole nursery phase, especially for those of NS1 that ratios showed values around 4, which were relatively higher than other Mediterranean oak seedlings (Tsakaldimi et al., 2005) and that trait was remained from the indoor cultivation phase (by means of significantly higher root fibrosity (FOLRs with d > 1 mm), R/S ratio and RDW) and be maintained for a year at the nursery. Thus, relatively large seedlings with greater nutrient reserves should be incorporated into Mediterranean restoration plantations (Villar-Salvador et al., 2008; Cuesta et al., 2010). By this fact, LED pre-cultivation further reinforces the predominance of better seedling quality traits than FL.

The DQI is considered an index of morphological development to predict seedling field performance (Dickson et al., 1960) and has been successfully used in several species (Luis et al., 2004; Marques et al., 2006). Consequently, an enhanced seedling quality index would enable better matching of seedlings to forest sites, by reducing the chance of regeneration delay and improving future growth of forest stands. Thus, all LED pre-cultivated *Q. ithaburensis* seedlings had significantly higher DQI than those of FL; even more those of NS1, mainly due to higher RCD and RDW accumulation, had significantly higher DQI than all LEDs after 6 months at the nursery. After 12 months at the nursery, FL seedlings showed significantly lower DQI compared to all LEDs except from G2, while seedlings of AP673L LED had the highest DQI of all treatments maybe due to lower plant height of the seedlings which nevertheless had high RCD.

Overall indoor cultivation of the tested broad-leaved species showed positively morphological and physiological adjustments after only 28 days of growing under different LED light qualities of continuous spectrum, compared to FL conventional light, reaffirming the reliance and sensitivity of the seedlings to the inherent need of light especially in early developmental stages. In addition, those light treatments triggered specific responses in the growth and morphology of the seedlings that were detectable even after the nursery transplanting and remained for a year; facts that could led us to accept both of our study’s hypothesis.

## Conclusion

These results confirm that many recognized advantages of LED lights as the mainly artificially light source for indoor cultivation of plants via conventional light sources like fluorescent lamps appear to hold true for the production of high quality seedlings of forest-tree species. All these attributes are further considered by the enhanced nursery performance of seedlings, which were the result of a remaining effect induced by the specific LEDs of continuous spectrum and were maintained even after a yearly exposure under natural light conditions. Each of the selected LED light qualities triggered various morphogenetic responses, which in turn could be useful for a targeted seedling production depending on the scope of a specific nursery manager or the specific needs of a regeneration site. High DQI could be a reliable prediction for a successful outplanting survival, especially in the harsh conditions of the Mediterranean region where the specific species has its natural range. However, information on the nursery production methods and quality of *Q. ithaburensis* seedlings and the growth and architectural development of these produced seedlings is limited. Consequently, further research is warranted to realize the full effects of LED lights on the morphology and physiology of forest tree species that are cultivated in order to obtain high quality planting stock material.

## Author Contributions

KR, TO, SS designed research. SS performed research. SS, TO, KR wrote the manuscript.

## Conflict of Interest Statement

The authors declare that the research was conducted in the absence of any commercial or financial relationships that could be construed as a potential conflict of interest.

The reviewer AM declared a past collaboration with one of the authors KR to the handling Editor, who ensured that the process met the standards of a fair and objective review.

## Abbreviations

CCI: chlorophyll content index
CD: cell density
DQI: Dickson’s quality index
FOLRs: first order lateral roots
F_v_/F_m_: maximum quantum efficiency of PSII photochemistry
LDW: leaf dry weight
LEDs: light-emitting diodes
PPFD: photosynthetic photon flux density
RCD: root collar density
RDW: root dry weight
RH: relative humidity
RL: root length
R/S: root:shoot ratio
SD: stomatal density
SDW: shoot dry weight
SH: shoot height
ShD: shoot diameter
SI(%): stomatal index (%)
